# Non-Adiabatically Tapered Optical Fiber Humidity Sensor with High Sensitivity and Temperature Compensation

**DOI:** 10.3390/s25144390

**Published:** 2025-07-14

**Authors:** Zijun Liang, Chao Wang, Yaqi Tang, Shoulin Jiang, Xianjie Zhong, Zhe Zhang, Rui Dai

**Affiliations:** 1 Future Technology School, Shenzhen Technology University, Shenzhen 518118, China; alex10280313@gmail.com (Z.L.); 13856038134@163.com (Y.T.); shoulinjiang@163.com (S.J.); awmc2837535441@gmail.com (X.Z.); drui12430@gmail.com (R.D.); 2 College of Engineering Physics, Shenzhen Technology University, Shenzhen 518118, China; zhangzhe@sztu.edu.cn

**Keywords:** optical fiber sensors, dispersion turning point, relative humidity sensor, dual-parameter sensors

## Abstract

We demonstrate an all-fiber, high-sensitivity, dual-parameter sensor for humidity and temperature. The sensor consists of a symmetrical, non-adiabatic, tapered, single-mode optical fiber, operating at the wavelength near the dispersion turning point, and a cascaded fiber Bragg grating (FBG) for temperature compensation. At one end of the fiber’s tapered region, part of the fundamental mode is coupled to a higher-order mode, and vice versa at the other end. Under the circumstances that the two modes have the same group index, the transmission spectrum would show an interference fringe with uneven dips. In the tapered region of the sensor, some of the light transmits to the air, so it is sensitive to changes in the refractive index caused by the ambient humidity. In the absence of moisture-sensitive materials, the humidity sensitivity of our sensor sample can reach −286 pm/%RH. In order to address the temperature and humidity crosstalk and achieve a dual-parameter measurement, we cascaded a humidity-insensitive FBG. In addition, the sensor has a good humidity stability and a response time of 0.26 s, which shows its potential in fields such as medical respiratory dynamic monitoring.

## 1. Introduction

Relative humidity is an important parameter in the fields of industrial production, agricultural cultivation, ecological protection, meteorological forecasting, and materials protection. Currently, electronic humidity sensors dominate the market, such as capacitive and resistive humidity sensors. However, after long-term exposure to high-humidity environments, electronic humidity sensors are prone to corrosion, rusting, and aging, which can be detrimental to the duration of their lifetime, their long-term stability, and their accuracy [1].

Fiber optic humidity sensors have attracted widespread research interest due to their light weight, small size, easy deployment, corrosion resistance, and electromagnetic interference resistance [2,3]. Optical fibers are inherently insensitive to humidity, but humidity sensing can be achieved by loading moisture-sensitive materials, such as polyvinyl alcohol and graphene oxide, onto a certain fiber-based sensing structure. A fiber humidity sensor can be made by coating the fiber Bragg grating (FBG) with hygroscopic materials, which would expand in a moist environment, pressure the FBG, and then cause a detectable shift in the Bragg wavelength. The typical sensitivity of a thermoplastic-polyimide-coded FBG humidity sensor is about 1.5 pm/%RH [4]. The humidity sensitivity would increase to 19 pm/%RH with a thicker polyimide coating of about 60 μm [5]. By applying a hygroscopic material to the bending-sensitive long-period fiber grating (LPFG), a humidity sensor can also be realized [5,6]. A spider dragline silk with super-contraction characteristics has been applied to a curvature-sensitive LPFG, and the resulting sensor can achieve a humidity sensitivity of −0.2039 nm/%RH [6]. Phase-sensitive fiber interferometers are also good platform for humidity sensing: a chitosan-film cascaded Fabry–Perot interferometric sensor based on the harmonic Vernier effect demonstrated a high humidity sensitivity of −83.77 nm/%RH [7]. When a section of Ti_3_C_2_T_x_ MXene-coated, tapered, no-core fiber with a waist diameter of 11.6 μm is added between two single mode fibers (SMFs), a tapered fiber interferometer is formed. Its humidity sensitivity can achieve a maximum of 685 pm/%RH within the range of 75~91%RH [8]. The use of hygroscopic sensitive materials is helpful to improve the sensitivity of fiber optic sensors. However, this material is prone to aging after long-term use, and its humidity response has high nonlinearity. In addition, these material-based humidity sensors typically have long response times of more than seconds [9].

Fiber humidity sensors without hygroscopic materials can circumvent these drawbacks, but they are often less sensitive. Such sensors are usually operated based on measuring humidity-induced changes in the refractive index (RI), so the key to improving their sensitivity is to use a fiber structure with high RI sensitivity [10,11,12]. Different fibers and optical structures have been applied to fibers for building RI-based humidity sensors. For example, the Sagnac loop interferometer, with an elliptical microfiber and a section of panda fiber [13]; the microfiber knot resonator, with a small bending radius [14]; the tapered PM-elliptical core fiber, bent into a U-shape [12]; and the Mach–Zehnder interferometer, with a section of photonic crystal fiber (PCF) sandwiched between two no-core fibers (NCFs) [11]. As far as we know, a hygroscopic-material-free fiber sensor, based on a Sagnac interferometer, shows the highest humidity sensitivity of 422.2 pm/%RH [13]. However, with regard to the humidity measurements, temperature crosstalk has the most significant effect on the sensor. Constructing a parameter matrix to measure both temperature and humidity at the same time is a common way to eliminate temperature crosstalk. Two separated optical microfiber couplers are combined to form a topological ring structure for RH and displacement dual-parameter sensing. By decoupling the sensitivity matrix, a humidity sensitivity of 180 pm/%RH can be achieved without any hygroscopic material [15]. A humidity and temperature dual-parameter sensor based on an intrinsic non-adiabatic tapered optical fiber was proposed. With an all-fiber structure, this sensor can achieve −47 pm/%RH sensing [16].

The dispersion turning point (DTP) fiber taper is a new dual-mode interferometric device made by tapering a section of common SMF down to a diameter of about 2 µm. This reduction in the core and cladding diameters within the tapered region alters the waveguide’s properties and enables the evanescent fields of the propagating modes to extend out of the microfiber. This evanescent field interacts directly with adjacent gas molecules. Consequently, modifications in the composition or the state of the ambient gaseous environment alter the optical path length difference experienced by the co-propagating modes. This results in a measurable change in the output interference spectrum. In the specially designed transition region of the DTP taper, a part of the transmitted light is converted to a higher-order transmission mode. Both modes then co-propagate along the waist, accumulating a relative phase difference. At a certain wavelength, known as the DTP, the group-effective RI difference between the two transmission modes is equal to zero. This unique condition leads to an extreme sensitivity of the accumulated phase difference to changes in the wavelength or the surrounding environment. The DTP fiber taper sensing structure has been applied to the measurement of a variety of physical quantities, for example, an axial strain sensitivity of 391.2 pm/με in the range of 0–300 με [17], a torsion sensitivity of 2.731 nm/(rad/m) [18], a salinity sensitivity of 6.138 nm/‰ in the range of 0–39.22‰ [19], and a temperature sensitivity of 6.44 nm/°C in the range of 30–70 °C [17]. Compared to the other micro- and nanofiber structure, the DTP taper has a larger diameter and also exhibits high sensitivity to environmental gas RI up to about 105 nm/RIU due to the dispersion turning effect at the wavelength near DTP [20]. The property of high RI sensitivity makes it a good candidate for making hygroscopic-material-free humidity sensors. D. Gao et al. fabricated an optical fiber mode interferometric humidity sensor with the sensitivity of −47 pm/%RH, and predicted a higher humidity sensitivity of the DTP taper humidity sensor [16]. However, the application of DTP tapers on humidity measurement has not been reported.

This paper presents a highly sensitive hygroscopic-material-free humidity sensor based on a DTP fiber taper. As the DTP device would also respond to environmental temperature with sensitivity of about several nm per centigrade [17], an FBG is connected in series with the DTP structure for temperature measurement, and the two form a humidity–temperature dual-parameter sensor. The sensor is made by applying a two-step fiber tapering method at the place adjacent to an FBG. By matrix calculations of the wavelength drift of FBG and DTP dips in the spectrum, the ambient temperature and humidity can be demodulated accurately. The stability and response time of the device are also measured.

## 2. Sensing Principle

**Figure 1:**
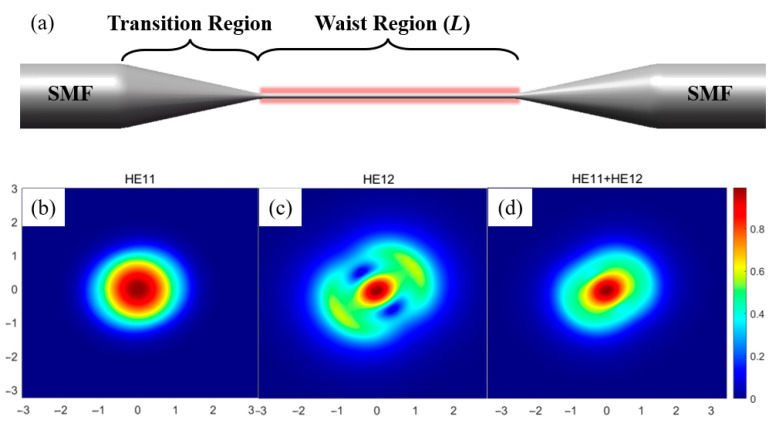
(**a**) Schematic diagram of the tapered optical fiber humidity sensor. The simulated mode patterns of the (**b**) HE_11_ (n_eff_ = 1.3743), (**c**) HE12 (n_eff_ = 1.0952), and (**d**) HE_11_ + HE_12_ of a 2.456 µm diameter taper waist.

A common optical fiber taper has a thin multimode waist region with both ends connected to SMFs through the transition regions, as shown in Figure 1a. For the adiabatic taper, the diameter of the transition region changes slowly along the fiber axial direction. The incident light from SMF will be converted to the fundamental mode in the waist region with low loss. In this case, there is no significant change in the transmission spectrum of the taper. For the non-adiabatic fiber taper with steep transition region, some energy of the incident light is coupled to the higher-order modes at the incident side and back to the fundamental mode at the other side. This results in interference fringes in the transmission spectrum of the tapered fiber. The intensity spectrum can be described by [21]:

(1) $$I=I_{1}+I_{2}+2\sqrt{I_{1}+I_{2}}\cos\Delta \varphi$$

$I_{1}$ and $I_{2}$ represent the light intensities of the fundamental and higher-order modes, respectively. Δφ is the phase difference between the fundamental mode and the higher-order mode, which can be expressed by the following formula:

$$\Delta \varphi =2\pi \frac{\Delta n_{eff}L}{\lambda_{N}}$$

*L* is the length of the waist region, and $\lambda_{N}$ is the wavelength of the *N*th dip on interference spectrum. When Δφ satisfies Δφ=(2N−1)π, i.e., $\Delta n_{eff}L$ is an even multiple of $\lambda_{N}$, the interference spectrum peaks appear. Conversely, if it is an odd multiple, the dips appear. The peaks and dips wavelength position can be controlled by designing the values of *L* and *n.*

To obtain a larger evanescent field and a clearer interference spectrum, the diameter of the taper waist is usually smaller than 3 µm [22]. The mode patterns and the effective RIs ($n_{eff}$) in a waist region were calculated by using the COMSOL Multiphysics 6.0. The simulation parameters were set as follows: the diameter of the cladding: 2.456 µm, the refractive index of the cladding: 14.378, and the wavelength is 1.55 µm. Figure 1b–d show the results of the fundamental mode HE_11_, higher-order mode HE_12_, and their combination, respectively. The high-order modes other than the HE_12_ mode cannot be effectively excited, because the geometries of their modes are quite different from the fundamental HE_11_ mode [23].

The RI fluctuations of the ambient air around the taper region will change the effective-RI-difference (${\Delta n}_{eff}$) of the transmission modes through the evanescent field, thus causing the output interference spectrum to drift. The RI sensitivity $S_{n}$ of the wavelength $\lambda_{k}$ at the dip can be expressed as [21]:

(2) $$S_{n}=\frac{{\partial \lambda }_{k}}{\partial n}=\frac{\lambda_{k}}{n_{g}^{HE11}-n_{g}^{HE12}}\frac{\partial ({\Delta n}_{eff})}{\partial n}=\frac{\lambda_{k}}{G}\frac{\partial ({\Delta n}_{eff})}{\partial n}$$

where $n_{g}$ is the group effective RI of mode and can be expressed by $n_{g}=n_{eff}-\lambda_{k}\partial (n_{eff})/\partial \lambda_{k}$. $\lambda_{k}$ refers to the wavelength of the *k*th dip on the interference spectrum. *G* is the difference in the *n_g_* of *HE*11 mode and *HE*12 mode. The value of *G* can be adjusted by changing the diameter of the taper. From Equation (2), when G approaches 0, the $S_{n}$ would approach positive infinity, which means that the taper possesses a huge RI sensitivity. This is the so-called DTP type spectrum, which would appear when the diameter of the waist region is about 2.2 µm [24].

The RI-sensitive DTP device can be used for humidity sensing as the air RI is affected by relative humidity (RH). At the standard environment condition (298.15 K, 1 atm, 300 ppm CO_2_), the relation between air RI ($n_{std}$) and RH can be expressed by the derivation of the Edlén and Jones’ equation [25,26]:

(3) $$n_{std}=1+\left(\frac{26,397.176}{Z}-4.296\times 10^{-4}\cdot e_{s}\cdot RH\right){\;\times \;10}^{-8}$$

where *Z* and $e_{s}$ are compressibility factor, and saturation water vapor pressure at a specific humidity and can be obtained by looking up the table. From Equation (3), when the air RH changes from 25% to 75% under standard conditions, *Z* is 0.99961 under 75%RH and 0.99966 under 25%RH, and *e_s_* is 3169 under both RHs. The resulting change in air RI is about $3.5\times 10^{-7}RIU/\%RH$.

In humidity sensing, both the RH value and the sensor structure are affected by ambient temperature; hence, temperature usually needs to be measured simultaneously with RH. The different spectral dips of the DTP device have different sensitivities for RI and temperature; however, their cross-sensitivity to RI and temperature is the same, so the dips cannot be used for dual-parameter sensing.

We propose cascading an FBG in the vicinity of DTP sensors for temperature and humidity dual-parameter sensing. The wavelength shift of the DTP device and the FBG as a function of ambient temperature and humidity changes can be represented by the matrix [27]:

(4) $$\left[\begin{array}{c} \Delta \lambda_{DTP}\\ \Delta \lambda_{FBG} \end{array}\right]=\left[\begin{array}{cc} S_{DTP-RH} & S_{DTP-T}\\ S_{FBG-RH} & S_{FBG-T} \end{array}\right]\left[\begin{array}{c} \Delta RH\\ \Delta T \end{array}\right]$$

(5) $$\left[\begin{array}{c} \Delta RH\\ \Delta T \end{array}\right]=\frac{1}{D}\left[\begin{array}{cc} S_{FBG-T} & -S_{DTP-T}\\ -S_{FBG-RH} & S_{DTP-RH} \end{array}\right]\left[\begin{array}{c} \Delta \lambda_{DTP}\\ \Delta \lambda_{FBG} \end{array}\right]$$

In Equation (4), $\Delta \lambda_{DTP}$ and $\Delta \lambda_{FBG}$ represent the wavelength shifts of the DTP dips and the FBG center wavelength. ΔRH and ΔT represent the changes in humidity and temperature; $S_{DTP-RH}$, $S_{DTP-T}$, $S_{FBG-RH}$, and $S_{FBG-T}$, respectively, represent the humidity and temperature sensitivities of the DTP dips and the FBG wavelength, which can be obtained by linear fitting of the experimental results. By converting Equation (4) into Equation (5), the change in humidity and temperature can be calculated by measuring the wavelength drift. The $D=|S_{DTP-RH}S_{FBG-T}-S_{DTP-T}S_{FBG-RH}|$ is the determinant value of the sensitivity coefficient matrix.

## 3. Sensor Fabrication and Test System

### 3.1. Fabrication of Cascaded FBG Tapered Fiber Optic Sensor

**Figure 2:**
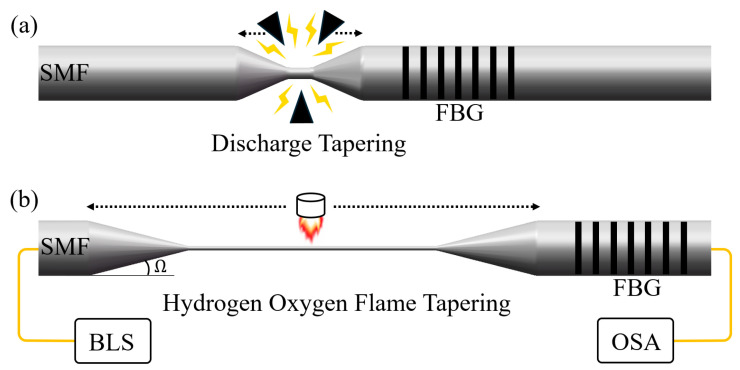
Schematic diagram of the two-step tapering process. (**a**) Pre-taper processing using an electrical discharge tapering machine. (**b**) Hydroxide flame tapering process. Ω is the taper angle of the transition zone.

**Figure 3:**
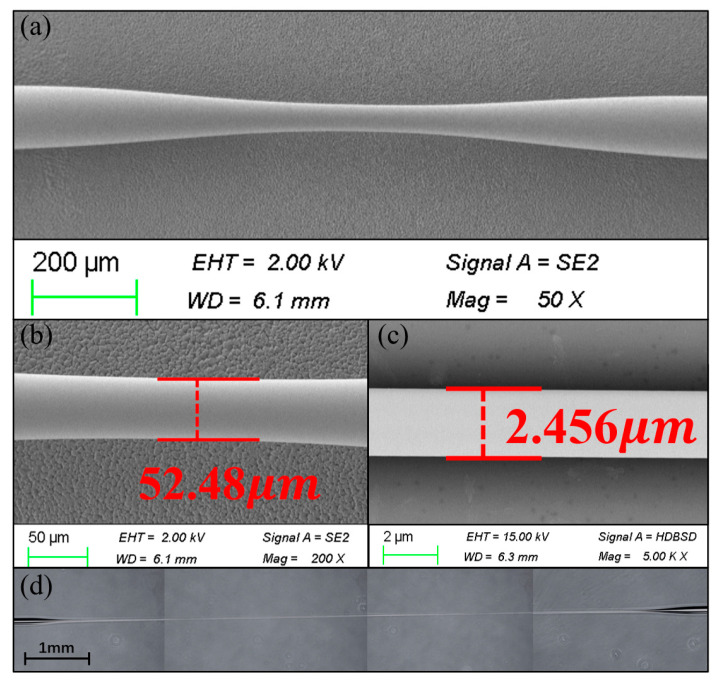
(**a**) SEM image of tapered zone after discharging tapering process. (**b**) SEM image of waist diameter after discharging tapering process. (**c**) SEM image of waist diameter after hydrogen oxygen flame tapering process. (**d**) Overall view of tapered region.

The DTP fiber taper is fabricated using a two-step tapering process, as Figure 2 illustrates. First, the coating layer of the SMF adjacent to the FBG region is peeled off for a 2 cm section. The section is then wiped with alcohol to clean the debris of coating material. Next, as Figure 2a shows, we use a three-electrode discharge tapering and splicing machine to make a short taper. Figure 3a shows the scanning electron microscope (SEM) side image of a short taper made using the parameters listed in Table 1. The waist diameter of the sample is about 52 μm, as shown in Figure 3b. This step helps generate a non-adiabatic transition during the subsequent drawing process.

To make a relatively long and uniform fiber taper, the hydroxide flame tapering process shown in Figure 2b is then required. The short taper is vacuum-fixed on the hydroxide taper machine. A 1 cm wide flame source heats the sample from above and scans it back and forth along the fiber. In this step, the transmission spectrum of the sample needs to be measured in real time with an optical spectrum analyzer (OSA, Yokogawa, Tokyo, Japan, AQ6370D) and a broadband light source (BLS, Golight, Shenzhen, China, OS-EB-L-D-1450-400-S-FA) to stop the machine in time when the DTP spectrum occurs at the wavelength near 1550 nm. Figure 3d shows a photomicrograph of a 1 cm long sample made by using the parameters in Table 2. The SEM image in Figure 3c shows that the waist of the sample is 2.456 µm in diameter.

**Figure 4:**
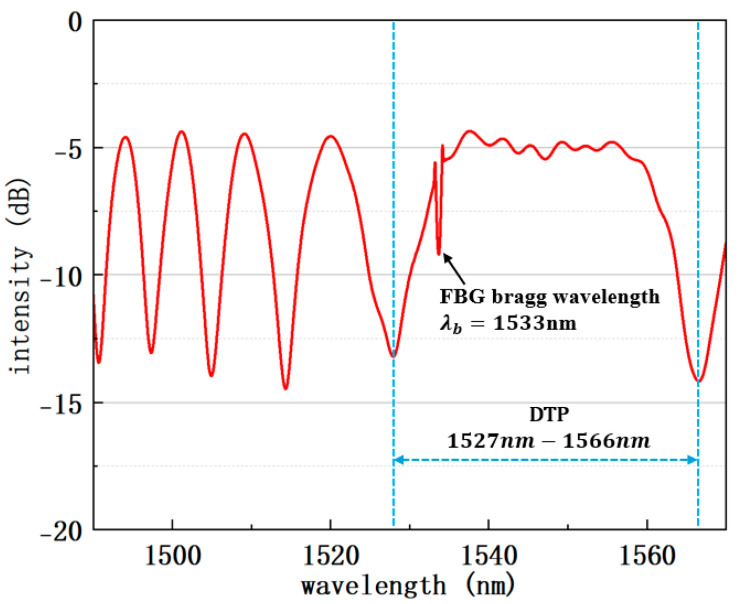
Transmission spectrum of the DTP-FBG structure shown with red line and DTP wavelength range shown with blue dashed lines.

The transmission spectrum of the sample is shown in Figure 4. The DTP appears at the wavelength of about 1546 nm. The dip at 1533 nm is the central wavelength of the FBG. Finally, the sample is fixed on a glass slide using UV-curing glue.

**Figure 5:**
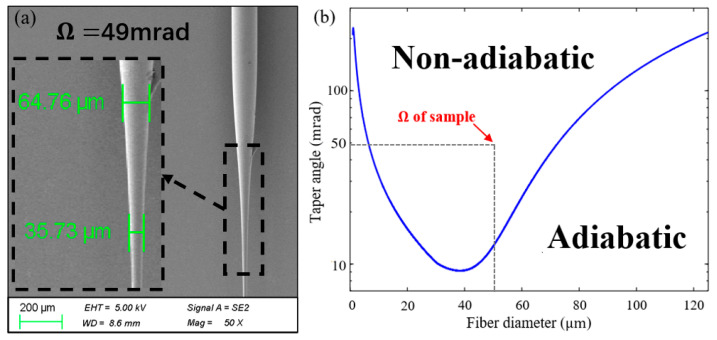
(**a**) SEM image of transition region after the flame tapering process. (**b**) Boundary line for taper angle as function of the diameter [28].

The non-adiabatic condition can be satisfied when characteristic taper length, $z_{t}$, is smaller than the characteristic beating length, $z_{b}$ [28].

(6) $$z_{t}=\frac{D}{tan(\Omega )}<z_{b}=\frac{\lambda }{n_{eff1}-n_{eff2}}$$

where *λ* is the free space wavelength, *D* is the diameter of the taper region, and $n_{eff1},\;n_{eff2}$ are the effective refractive indexes of both modes. Satisfying the non-adiabatic condition in the tapered transition zone is necessary for the effective excitation and selection of high-order modes and spectrum contrast enhancement. Based on the SEM photographs of the transition zone in Figure 5a, the taper angle *Ω* of the sample is estimated to be about 49 mrad in the transition region diameter from 35 µm to 64 µm. As shown in Figure 5b, the taper angle of the sample made by the two-step method is far away from the adiabatic region, so the high-order mode in the transition zone can be efficiently converted to the fundamental mode.

### 3.2. Temperature and Humidity Testing System

**Figure 6:**
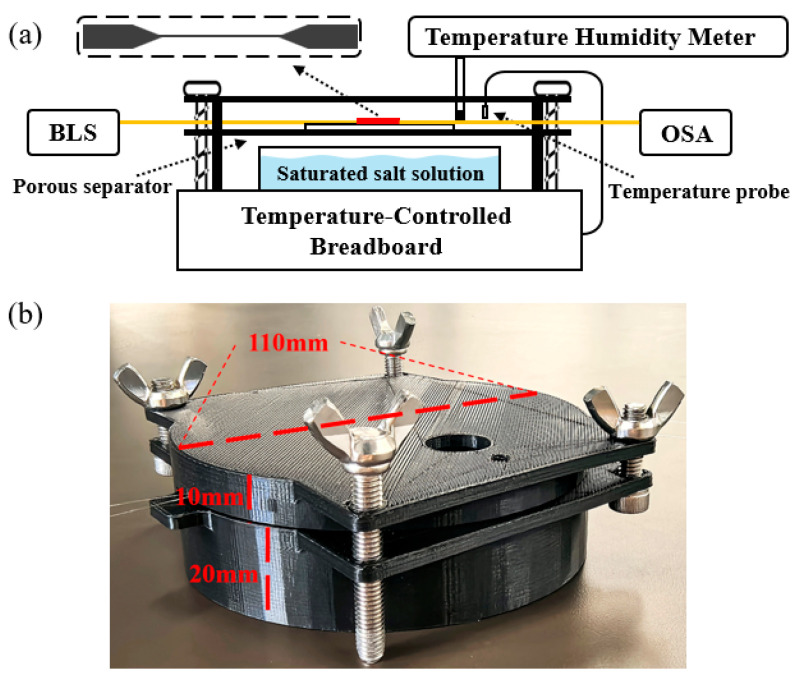
(**a**) Humidity and temperature testing system. (**b**) Photograph of humidity chamber assembly.

The performance of the FBG-integrated DTP humidity sensor was measured in a system, as Figure 6a shows. The sensor sample was fixed on the porous separator in a 3D-printed chamber, as Figure 6b depicts. The volume of this closed chamber is about 200 mL, and its upper and lower parts communicate internally. The in-chamber humidity was set by different types of supersaturated salt solutions and reduced by desiccant. The salt solutions of MgCl_2_, NaBr, NaCl, and KCl were used in our experiment to generate humidity levels of 33%RH, 57%RH, 75%RH, and 85%RH, respectively. The temperature in the chamber is closed-loop controlled by the temperature controller with external probe (Thorlabs, Newton, NJ, USA, PTC1/M). The actual temperature and humidity around the sample are monitored with a commercial temperature humidity meter (THM, Fluke, Shanghai, China, 971). The transmission spectra of the sample are measured using the OSA and the BLS as shown in Figure 2b.

## 4. Experiment Result and Discussion

### 4.1. Humidity and Temperature Dual-Parameter Measurement

**Figure 7:**
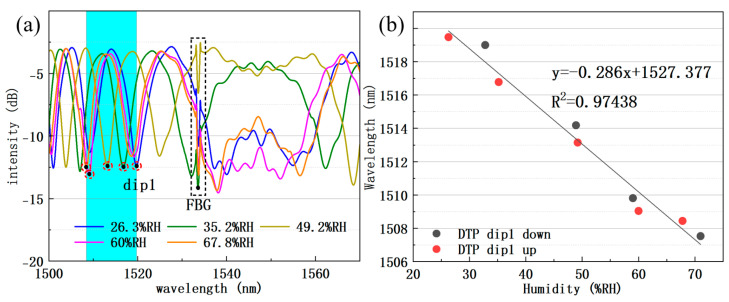
(**a**) DTP interference spectra in different ambient RH at 25 °C; the drift range of dip1 is shown in the blue area. (**b**) The wavelength of dip1 as a function of chamber RH from linear fit.

The humidity experiments were conducted with the chamber temperature locked at 25 °C. After the THM reading stabilized, the humidity value and interference spectrum were synchronously recorded. We changed the type of saturated salt solution to adjust the RH value inside the system. Figure 7a shows the drift of the interference spectrum with different chamber humidity levels. Then, we repeated the experiment with a downward humidity adjustment from 62%RH to 25%RH. As shown in Figure 7b, the dip1 wavelength shows good linearity with RH value throughout both humidity rise and fall experiments. The RH sensitivity is about −286 pm/%RH within the 30~72%RH range. As there is no evanescent field in the FBG region, the FBG dip wavelength did not change significantly during the entire humidity experiment [29].

**Figure 8:**
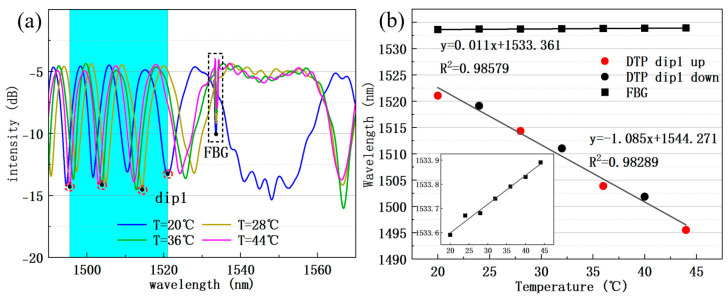
(**a**) DTP interference spectra at different temperatures under a fixed humidity environment; the drift range of dip1 is shown in the blue area. (**b**) Temperature sensitivity of the FBG and the dip1 compared for temperature increasing and decreasing runs.

We also measured the temperature response of the sensor by placing excess desiccant in the lower chamber to keep humidity constant and adjusting the heating stage to vary temperature from 20 to 44 °C in 4 °C increments. The interferometric dip1 shows −1.085 nm/°C sensitivity, while the FBG exhibits 0.011 nm/°C, as shown in Figure 8a,b. Due to different sensing principles, FBG and dip1 exhibit opposite drift directions as temperature increases.

By substituting experimental humidity and temperature sensitivity results into Equation (5), the matrix relationship between the wavelength changes of dip1 and FBG and the changes in temperature and humidity of the sensor sample can be obtained:

(7) $$\left[\begin{array}{c} \Delta RH\\ \Delta T \end{array}\right]=\frac{1}{3.146\times 10^{-3}}\left[\begin{array}{cc} 0.011 & 1.085\\ 0 & -0.286 \end{array}\right]\left[\begin{array}{c} \Delta \lambda_{dip1}\\ \Delta \lambda_{FBG} \end{array}\right]$$

According to the equation, humidity and temperature values can be calculated by measuring the wavelength shifts of dip1 and FBG. Although the structure of the micro-nano fiber made of silica is stable at high temperatures above 100 °C, when a dip in the interference spectrum of the DTP tapered fiber drifts over a large temperature range, it shows nonlinear drift at high temperature, directly affecting measurement accuracy when using fixed matrix parameters. This could be solved by shifting the measurement dip when temperature change is high and the blue shift of the target dip wavelength is large. The FBG dip can monitor temperature and assist the target dip changing. Nevertheless, within a small temperature range, for example, 20–44 °C in our experiment, as shown in Figure 8, the wavelength drift of dip1 ranges from 1495 nm to 1521 nm and shows good linearity over the 24 °C temperature range.

### 4.2. Stability and Response Time

**Figure 9:**
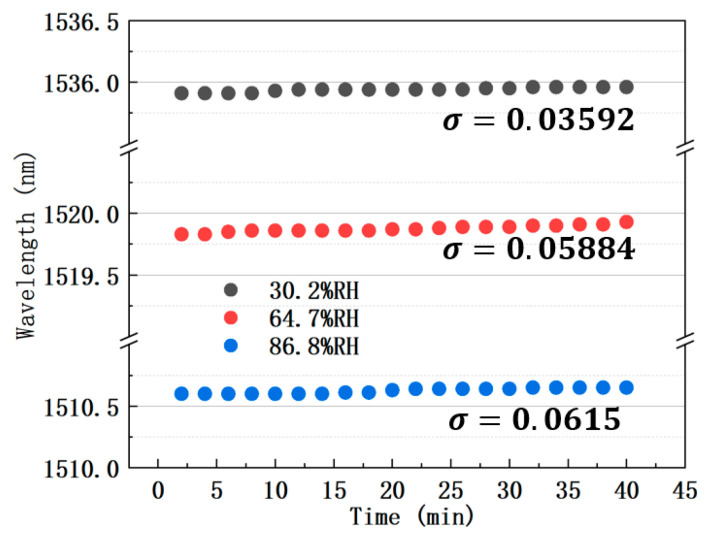
Stability of sample dip wavelength at three different RH.

The stability experiment was conducted at three different RH levels of 30.2%, 64.7%, and 86.8%, and a fixed temperature of 25 °C. Transmission spectra were recorded every 120 s over 40 min. As shown in Figure 9, the standard deviation σ of dip wavelength at 30.2%RH, 64.7%RH, and 86.8%RH was 0.03592 nm, 0.05884 nm, and 0.0615 nm, respectively. By converting the dip wavelength fluctuation range (σ) at these humidity values into humidity fluctuation range using humidity sensitivity (−286 pm/%RH), the worst relative error of RH in 40 min obtained by dividing with the corresponding ambient humidity value is 0.4% at 30.2%RH.

**Figure 10:**
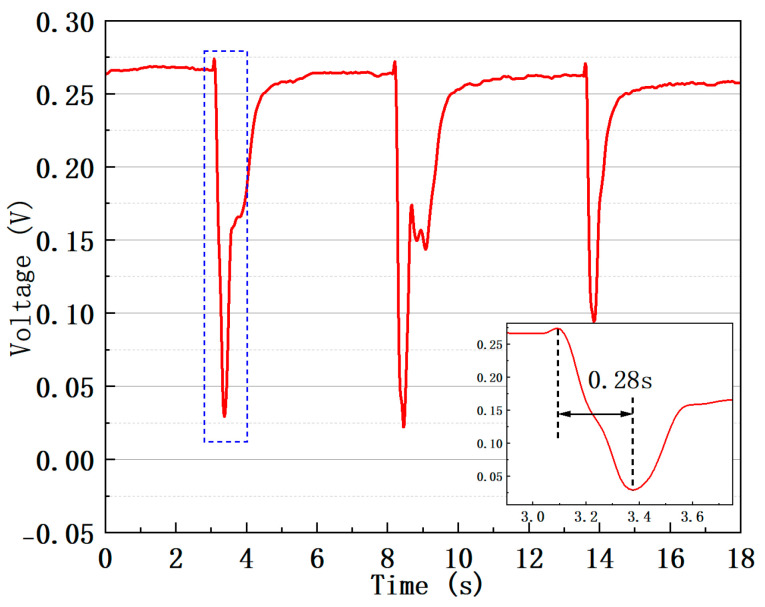
The sensor’s response to sudden changes in humidity. Inset: The response of the sensor to the first humidity increases in the blue dashed square.

To measure the sensor’s response time to humidity, we replaced the BLS in the Figure 6a system with a tunable narrowband laser (Santec, Komaki, Japan, TSL-710), and the OSA with a photodetector (Newport, Newport, RI, USA, 2011FC-M) and an oscilloscope (Tektronix, Shanghai, China, TBS 1000x). In the chamber, the temperature was fixed at 25 °C. The wavelength of the narrowband light laser was initially set to 1540 nm, which corresponds to a wavelength at half the peak intensity of the interference spectrum. Then, humidity changes quickly by exhalating air into the chamber. The ambient humidity rises sharply from 45%RH to 95%RH. Figure 10 shows the decrease in transmitted light intensity caused by three exhalations. The average time of the three signals from the initial value to the minimum is about 0.26 s. The recovery time is about 3.25 s. This response time is comparable to that of other hygroscopic-material-free optical humidity sensors.

### 4.3. Comparison and Discussion

We show a comprehensive comparison analysis of our DTP optical fiber taper with other hygroscopic-material-free optical fiber humidity sensors in Table 3. DTP optical fiber taper’s humidity sensitivity is slightly lower to the sensitivity of the SI combined with Panda fiber [13], but compared with the expensive Panda fiber and complex SI manufacturing process, DTP optical fiber taper has the advantages of low cost and simple process. Its stability and response time are close to other sensors, and dual parameters can also be measured simultaneously.

## 5. Summary and Conclusions

This study introduces an all-fiber sensor for simultaneous measurement of humidity and temperature. The sensor consists of a non-adiabatic fiber taper and an FBG connected in series. The taper is made using a two-step tapering method. By controlling the shape of the transition region and the diameter of the waist region, the taper can exhibit ultra-high RI sensitivity in the DTP region of its transmission spectrum. As both the environmental humidity and the temperature would affect the RI of the taper, a temperature-sensitive FBG is used to assist the parameters’ decoupling. The sensor sample with humidity sensitivity of −286 pm/%RH and temperature sensitivity of −1.085 nm/°C was realized. The humidity response time of the sensor is about 0.26 s. This hygroscopic-material-free sensor possesses the advantages of low cost, simple fabrication process, and short response time. The sensor has good potential in respiratory dynamic monitoring and some other medical, industrial, and agricultural applications.

## Figures and Tables

**Table 1:** Discharging tapering parameter (3SAE, U.S.A., LDS 2.5).

Start Speed (μm /s)	Sweep Speed (μm /s)	Waist Length (μm)	Start Power	Waist Power
50	600	500	400	450

**Table 2:** Flame tapering parameter (Coupler, Jinan, China, AFBT8000).

Step	Stretching Length (μm)	Fire Height (mm)	Stretching Speed (μm/s)	Hydrogen Flow Rate (sccm)	Sweep Speed (μm/s)	Sweep Distance (μm)
Step1	0–4000	3.011	70	180	/	/
Step2	4000–10,000	3.011	90	150	2000	6000

**Table 3:** Performance comparison analysis.

Sensors	Humidity Sensitivity	Range	Cost	Stability	Response Time	Citation
Panda fiber-SI	−0.422 nm/%RH	25–70%RH	high	0.024 dB in 1 min	0.06 s	[13]
Tapered microfiber	0.18 mW/%RH	28–90%RH	low	3.66% in 3 h	0.8 s	[14]
Microfiber knot resonator	0.010 nm%RH	35–95%RH	low	0.28 dB in 3 h	1.55 s	[14]
U-shaped-taper-MI	0.115 nm/%RH	30–90%RH	low	0.4% in 3 h	/	[12]
NCF-PCF-NCF-taper-MZI	0.02 nm/%RH	30–70%RH	high	2.7% in 2 h	/	[11]
DTP-FBG taper	−0.286 nm/%RH	25–86%RH	low	0.4% in 40 min	0.26 s	This work

## Data Availability

The original contributions presented in this study are included in the article. Further inquiries can be directed to the corresponding author(s).
